# Supplementary material for: Tersicoccus phoenicis (Actinobacteria), a spacecraft clean room isolate, exhibits dormancy
Source: Microbiol Spectr. 2025 Aug 11;13(9):e01692-25. doi: 10.1128/spectrum.01692-25 (PMC12403811; doi:10.1128/spectrum.01692-25)
Supplement: Supplemental figures — Fig. S1 to S8. [file spectrum.01692-25-s0001.docx]

**
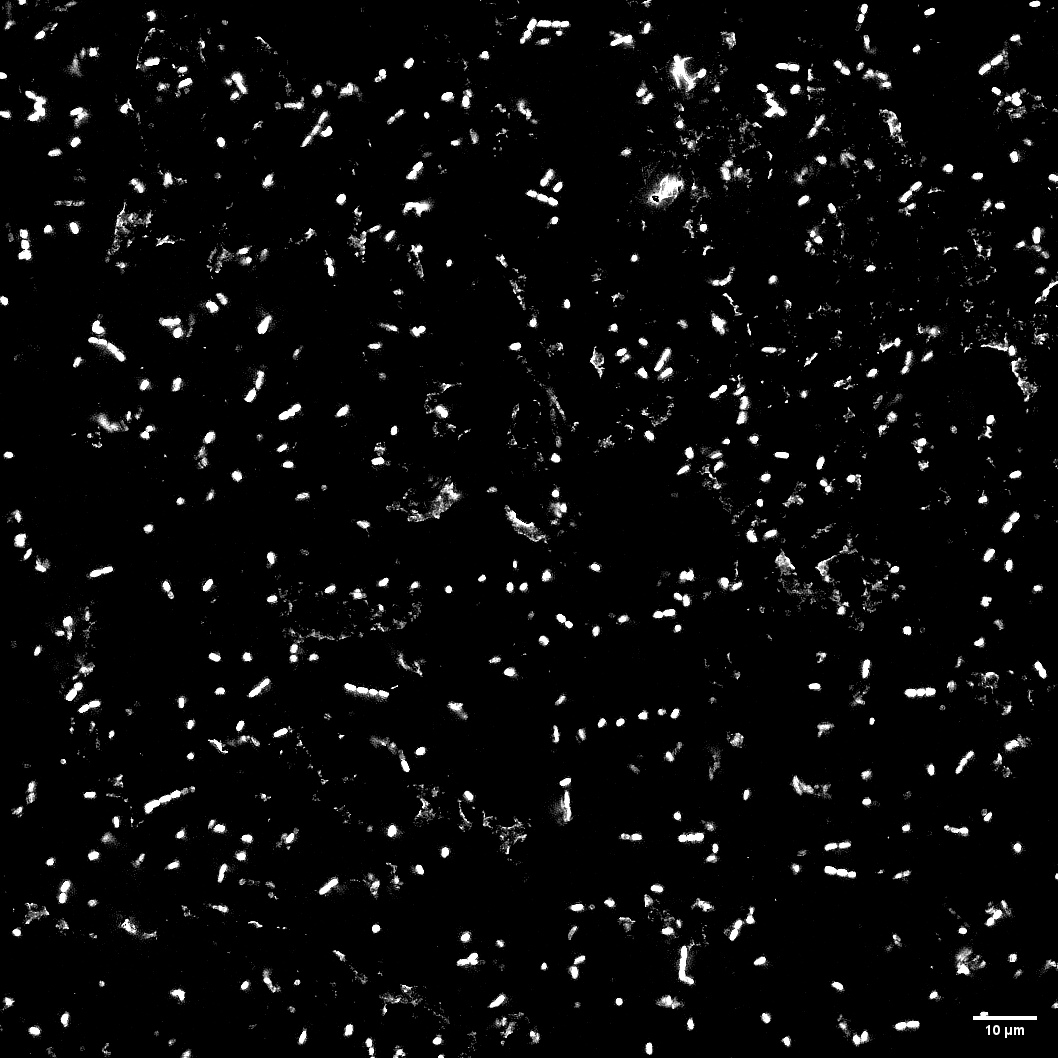
**

**Figure S1 (A).** Micrographs of *Tersicoccus phoenicis* in exponential growth in acetate minimal media (AMM). The images were captured on a Tomocube HT-X1 quantitative phase imaging microscope. Scale bar = 10$\mu$m. Notably, no morphological differences were evident between the exponential and dormant states. This observation supports the presence of a viable but non-culturable (VBNC) phenotype, rather than a morphological transformation.

**
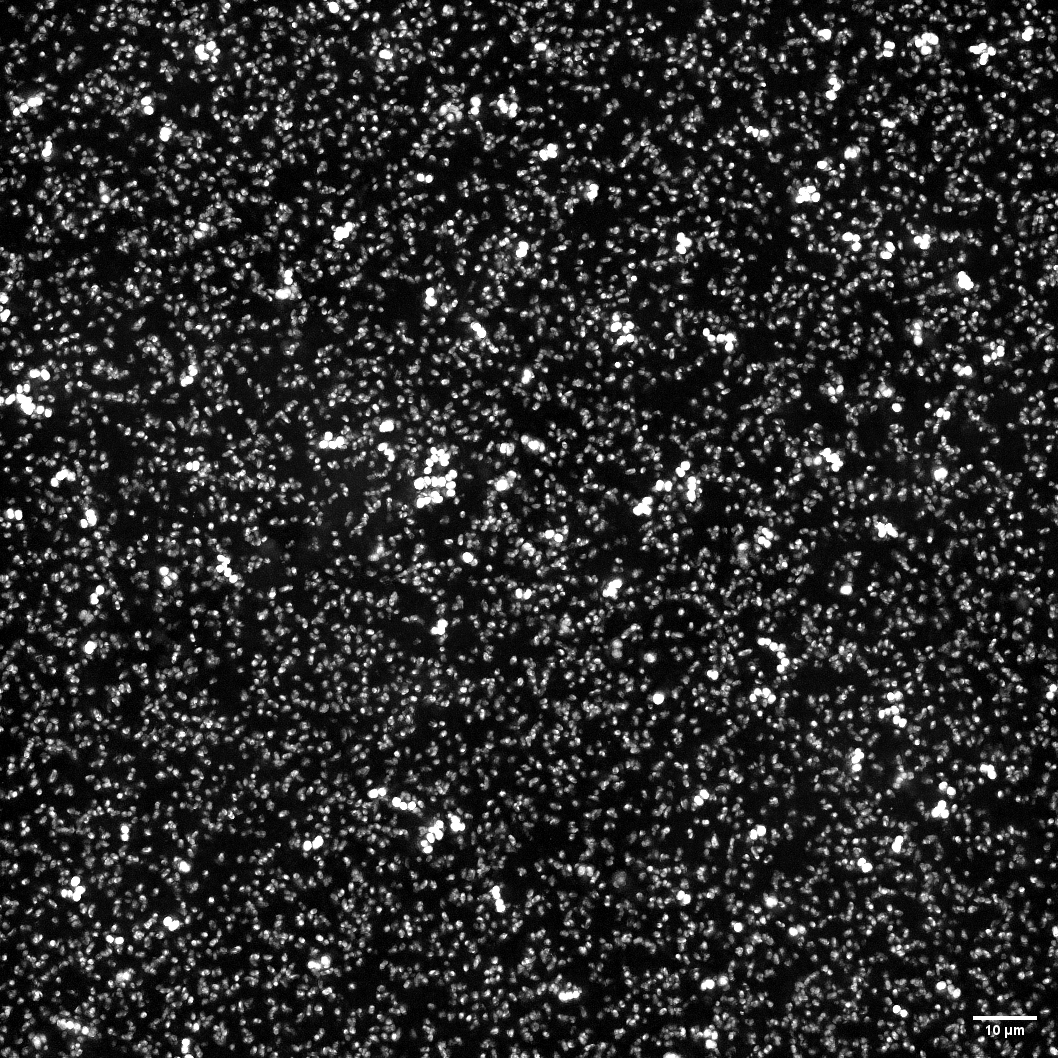
**

**Figure S1 (B).** Micrograph of *Tersicoccus phoenicis* in the dormant state after 10 days of growth in acetate minimal media (AMM). The images were captured on a Tomocube HT-X1 quantitative phase imaging microscope. Scale bar = 10$\mu$m. Notably, no morphological differences were evident between the exponential and dormant states. This observation supports the presence of a viable but non-culturable (VBNC) phenotype, rather than a morphological transformation.


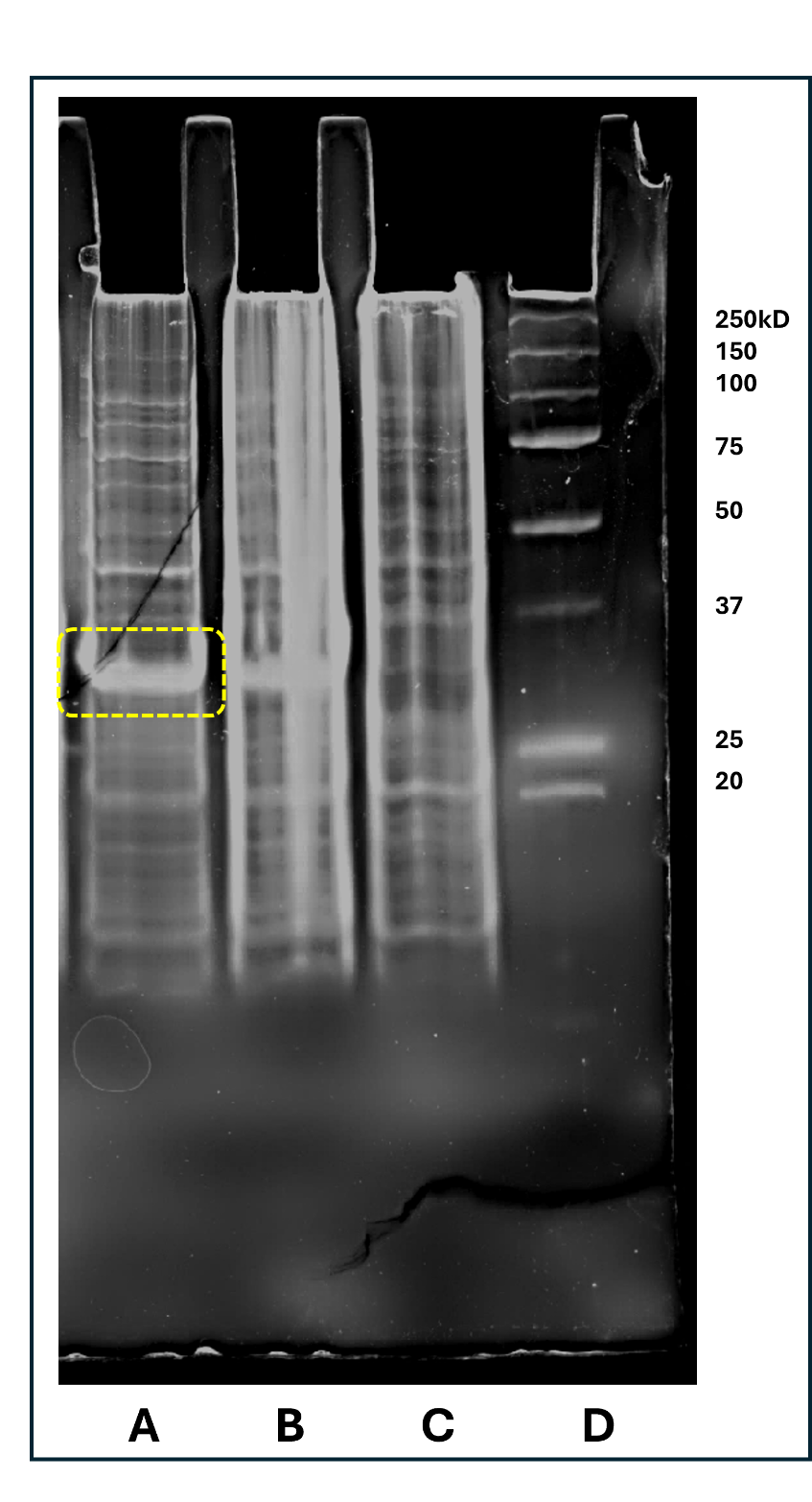


**Figure S2.** Lysate of *E. coli* overexpressing the Rpf gene from *M. luteus* was centrifuged, and the supernatant was passed through a 0.2 micron filter and loaded on a 12% SDS-PAGE gel (MOPS buffer). The gel was run for 2.5 hrs at 90V Voltage, stained with Coomassie blue and destained with 10% methanol solution. Lanes A and B: lysate from the induced cells showing overexpressed Rpf showing the putative protein band marked in an yellow box, lane B: lysate from the uninduced cells that do not show the band with the same intensity. Lane D: Protein molecular weight marker (BioRad).


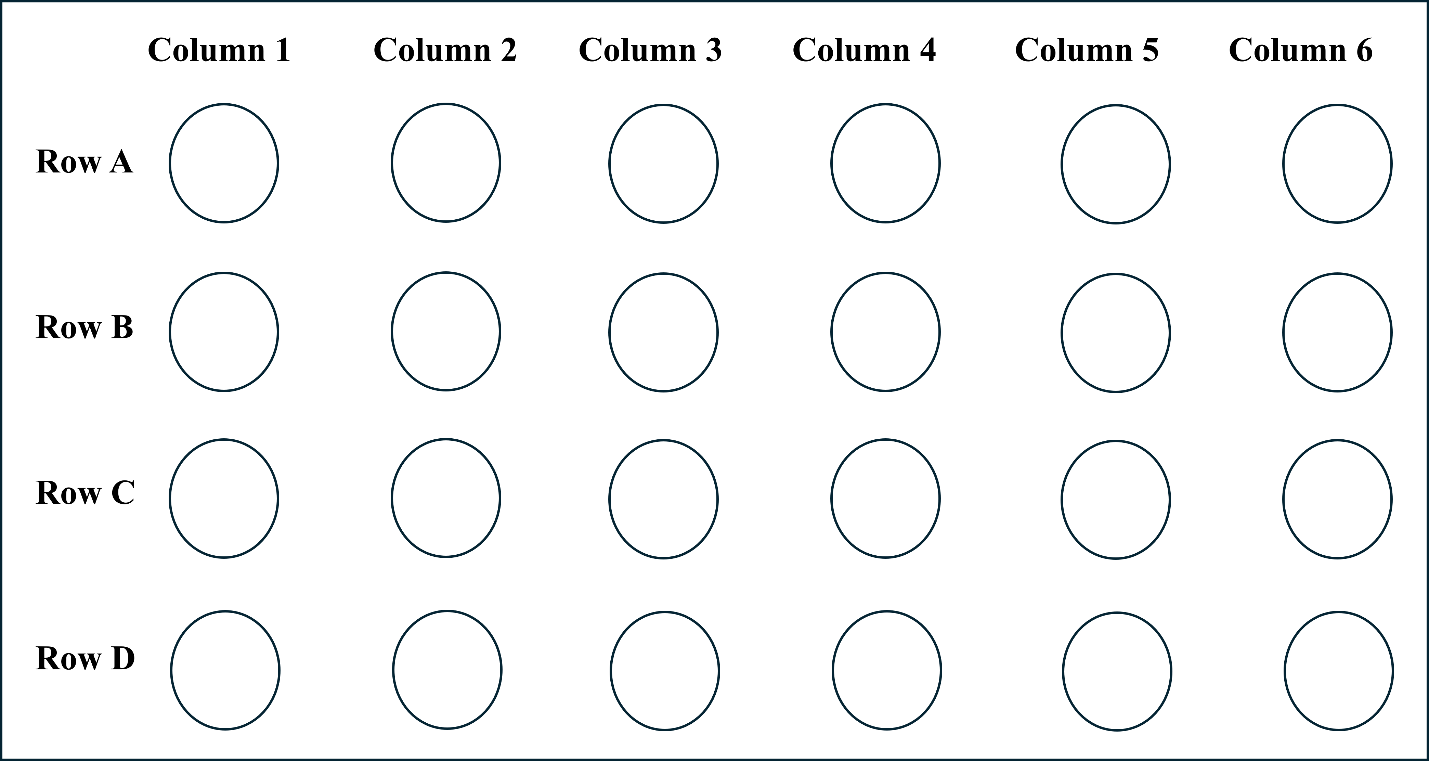


**Figure S3.** Layout of the 24-well plate used for dormancy revival screening in LB and AMM (acetate minimal media). One of the columns had undiluted lysate (of *E. coli* overexpressing the Rpf gene from *M. luteus*), another had no lysate (negative control), while the other four columns had 10-fold serial dilutions of lysate. Cells were inoculated into each well containing 1ml of either LB or AMM. Growth was conducted and measured every 15 minutes using a Tecan SpectraFluor Plus instrument. Continuous shaking was maintained between successive readings.

**
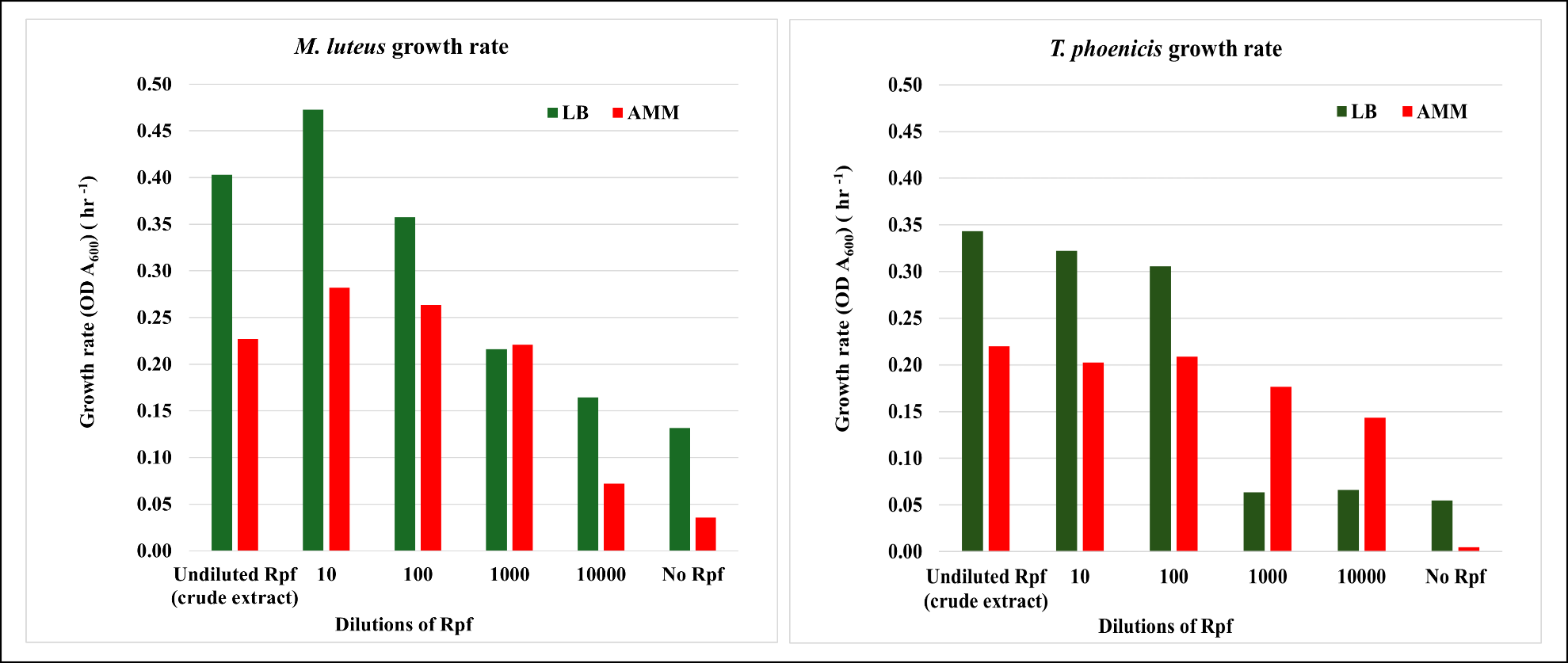
**

**Figure S4.** The growth rate(s) of dormant cells of *M. luteus* and *T. phoenicis* in LB (Luria broth) and AMM (acetate minimal media). The undiluted crude lysate (of *E. coli* overexpressing the Rpf gene from *M. luteus*), containing the Rpf ((~1µM (27.5 µg/ml)) and the crude extract diluted 10, 100, 1000, and 10000 times was used to evaluate their effect on the growth. Growth rate was directly proportional to the Rpf (lysate) levels in the growth media. Dormant cells of *T. phoenicis* in particular showed negligible growth in the absence of Rpf (lysate) in AMM.

**A**


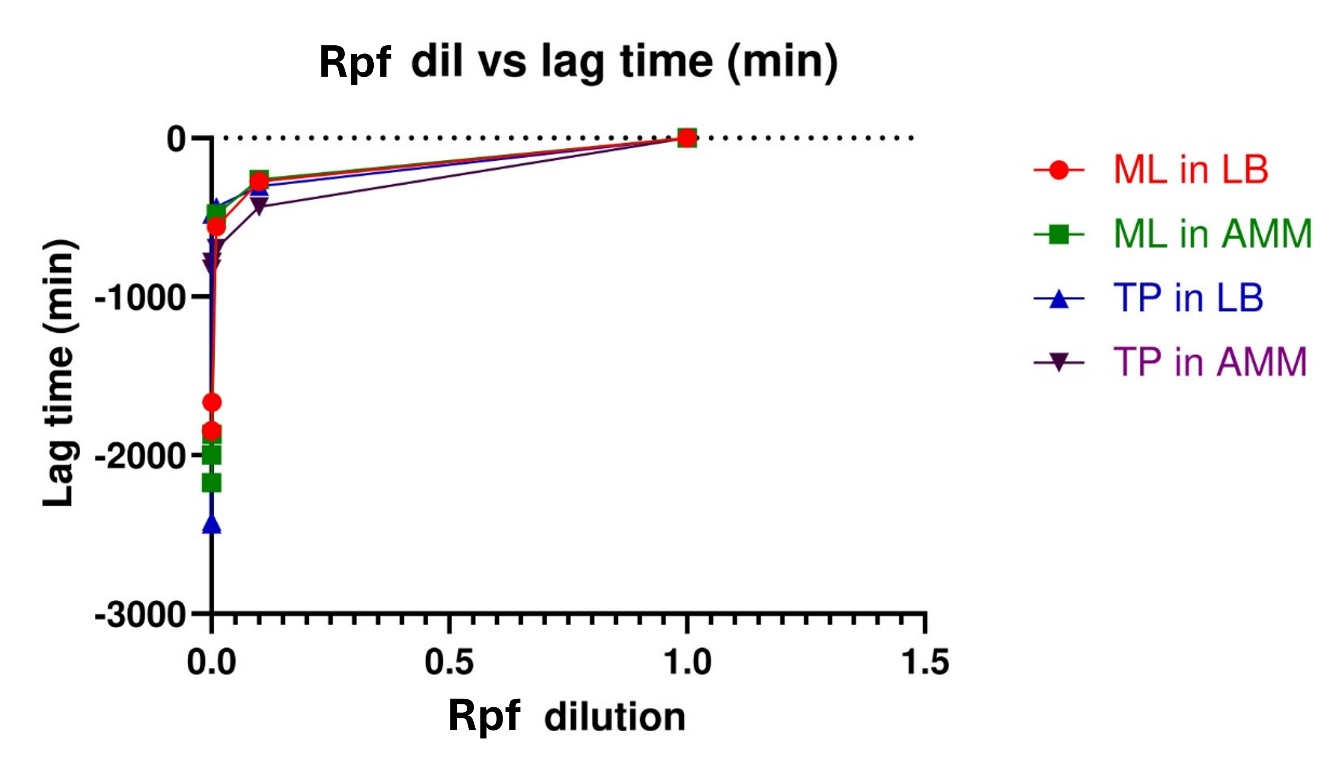


**Figure S5.** **(A)** Lag times (negative) plotted vs the Rpf concentration (lysate obtained from *E. coli* overexpressing the Rpf gene from *M. luteus*).

**B**


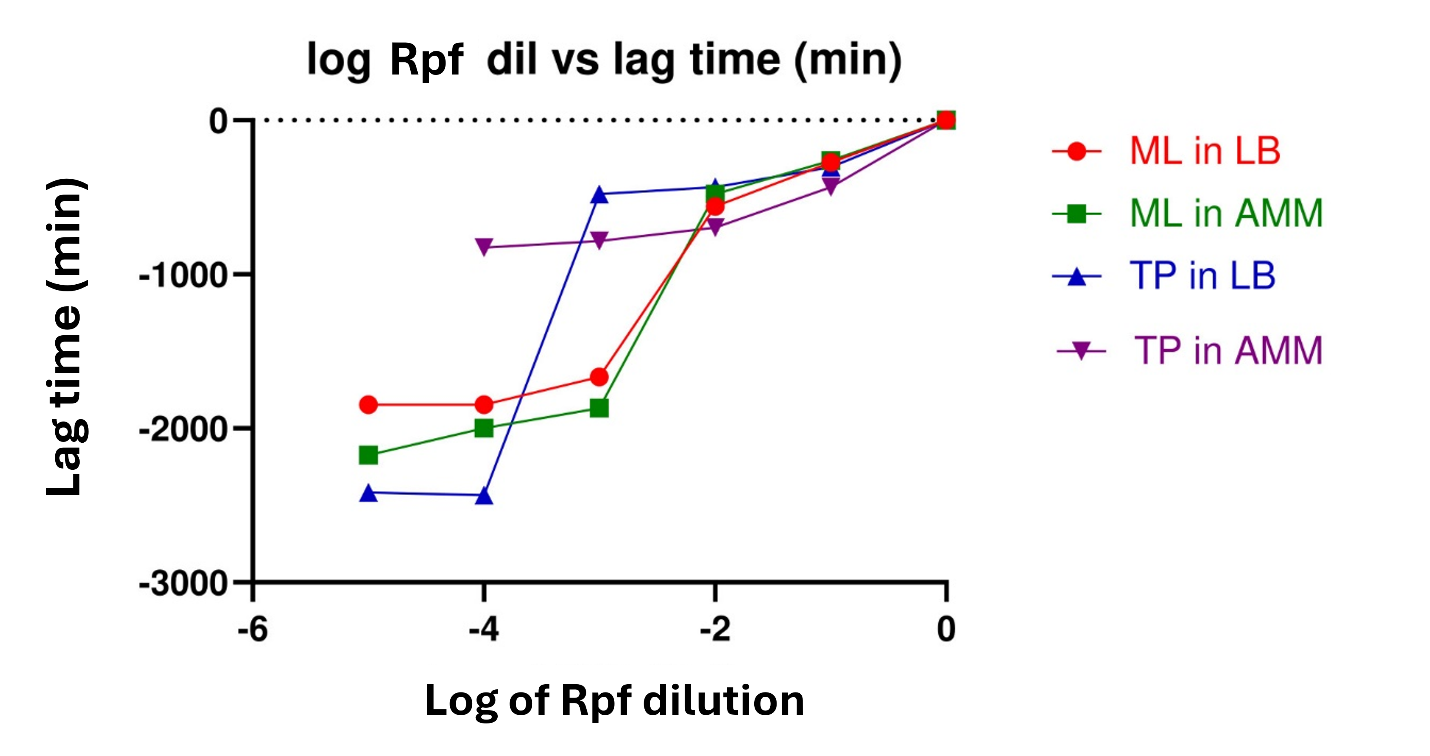


**Figure S5.** **(B)** Lag times (negative) plotted vs the log Rpf (lysate) concentration. The undiluted Rpf (lysate) concentration used in the experiment(s) was ~1µM (27.5 µg/ml). The absolute growth time of the fastest growing well in each row (highest Rpf (lysate) concentration) was set to a relative zero time and the relative lag times were determined from this initial relative time for the other Rpf (lysate) concentrations. The negative of the time was plotted to indicate the increased lag. ML - *Micrococcus luteus*; TP- *Tersicoccus phoenicis*.


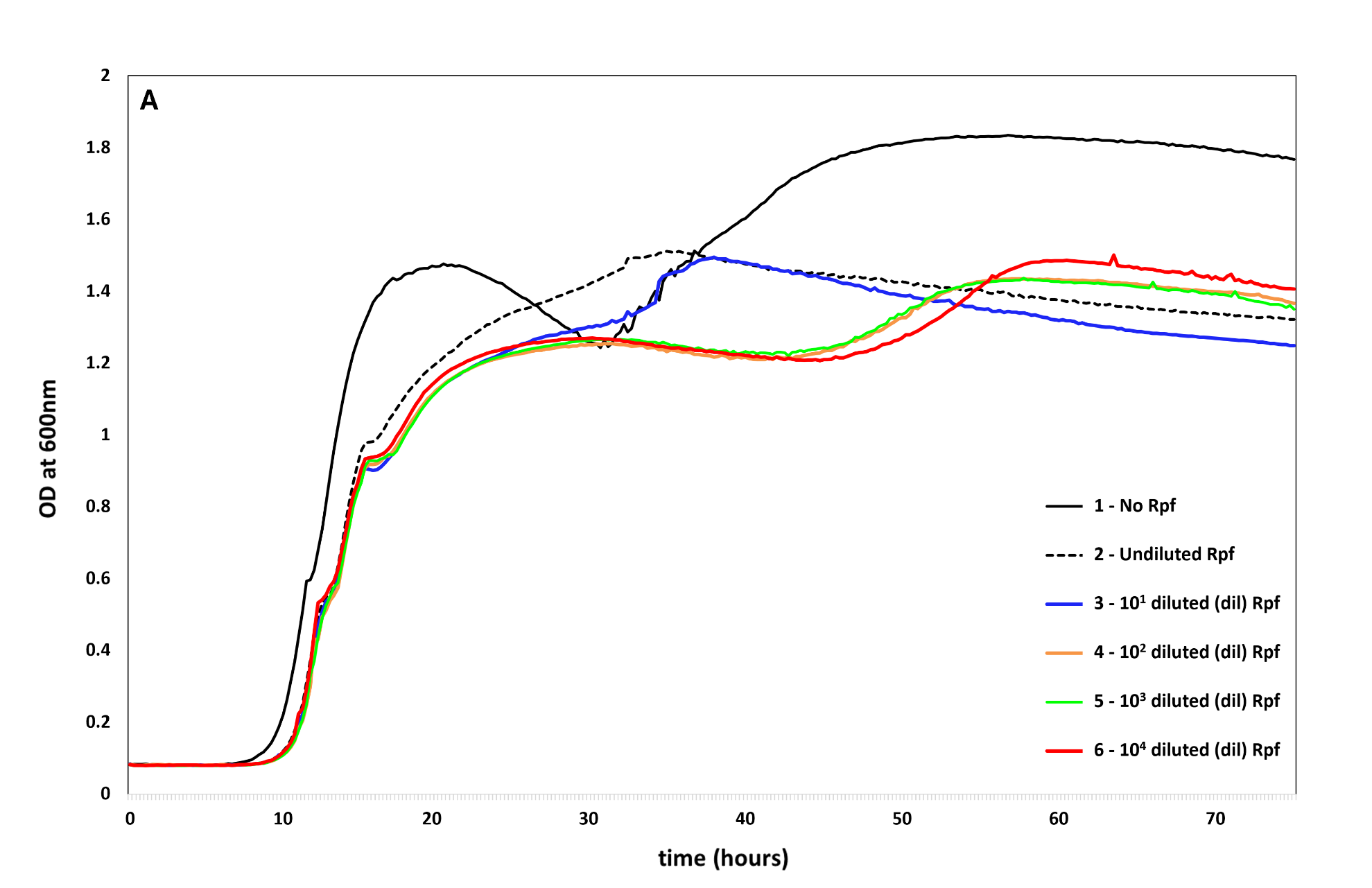


**Figure S6.** The effects of Rpf (lysate) (lysate of *E. coli* overexpressing the Rpf (lysate) gene from *M. luteus*) on the growth curves of *T. phoenicis* in LB media in triplicates. **(A).** Wells contained inoculum of growing cells of *T. phoenicis*. Growth was conducted and measured every 15 minutes in a 24-well (4×6) microtiter plate with 1mL of medium per well using a Tecan SpectraFluor Plus instrument. Continuous shaking was maintained between successive readings.

**
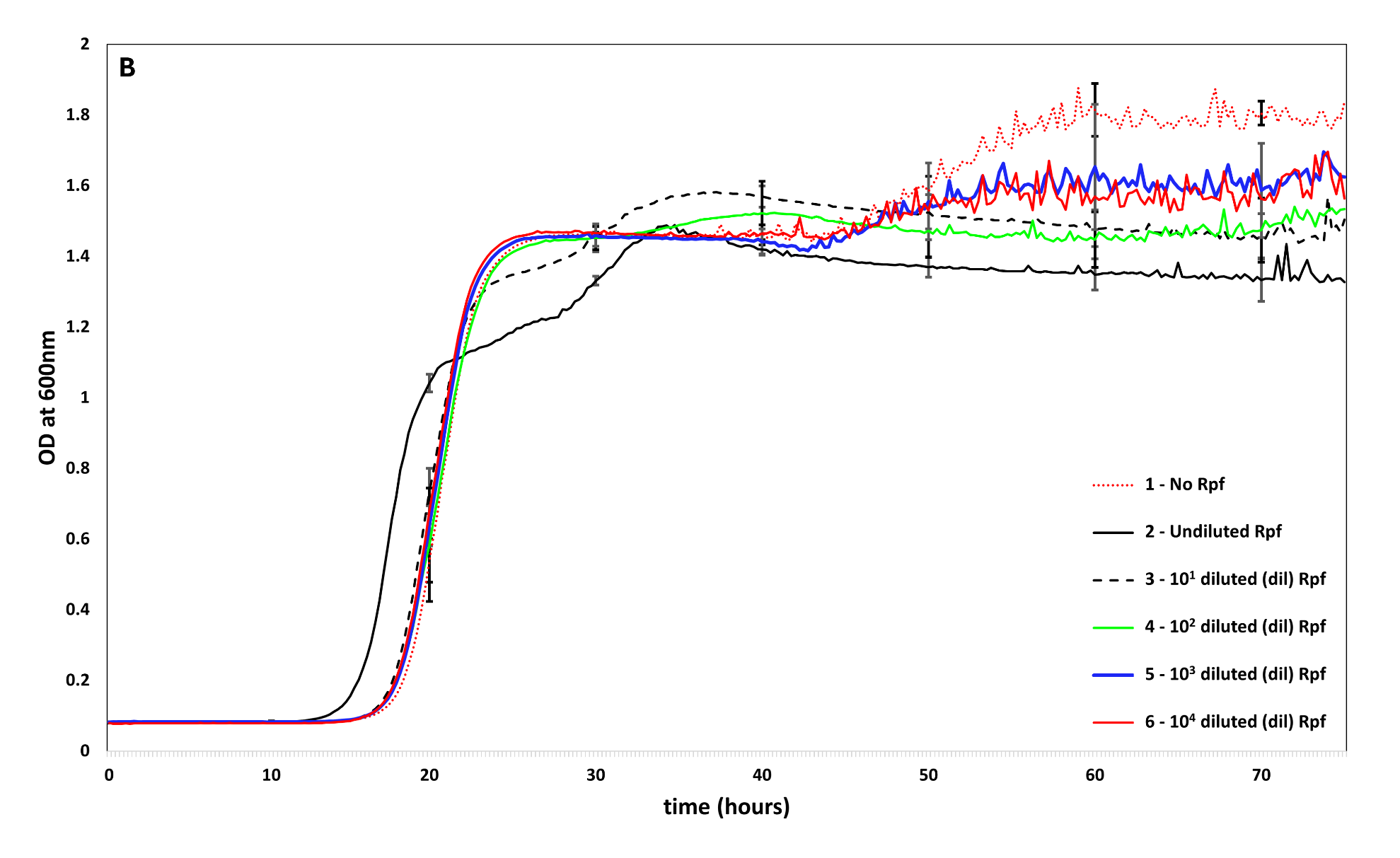
**

**Figure S6. B**. Growth curve(s) of dormant cells of *T. phoenicis* (obtained following growth in Acetate Minimal Media (AMM)) with growth in triplicates*.* In all the rows, column 1 had no Rpf (lysate), column 2 had 10 µl of undiluted Rpf (lysate) ((~1µM (27.5 µg/ml)), while column 3-6 were serially diluted. Line graph showing the average values of datasets from the replicates (n=3) across time. At each time point, the mean of the three datasets was computed, and the standard deviation was calculated to represent the variability. Error bars indicate standard deviation. Growth was conducted and measured every 15 minutes in a 24-well (4×6) microtiter plate with 1mL of medium per well using a Tecan SpectraFluor Plus instrument. Continuous shaking was maintained between successive readings.

**
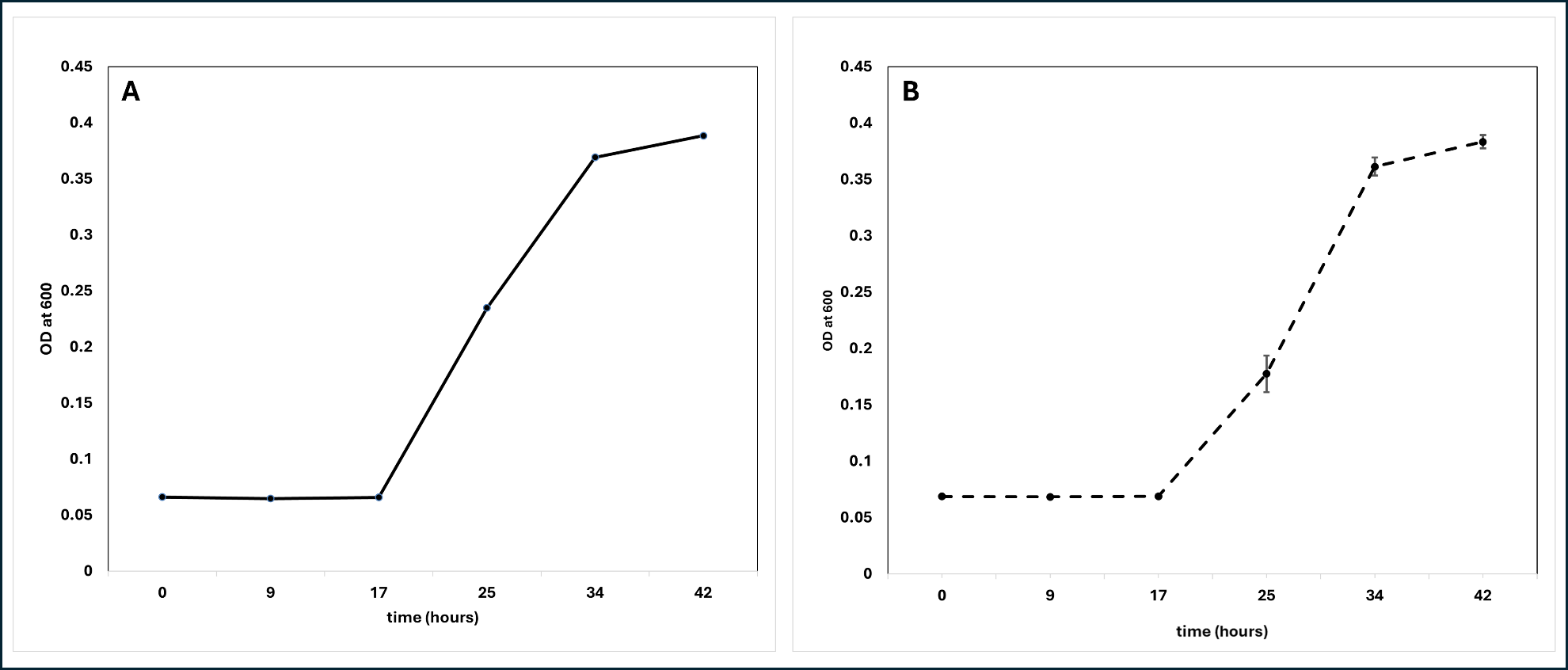
**

**Figure S7.** **A:** Growth curve of cells of T. phoenicis (from logarithmic phase) resuspended in AMM. **B**: Growth curve(s) of 48 hrs (2 days) air-dried (dormant) cells of T. phoenicis resuspended in AMM in triplicates. Line graph showing the average values of datasets from the replicates (n=3) across time. At each time point, the mean of the three datasets was computed, and the standard deviation was calculated to represent the variability. Error bars indicate standard deviation. Growth was conducted and measured every 15 minutes in a 24-well (4×6) microtiter plate with 1mL of medium per well using a Tecan SpectraFluor Plus instrument. Continuous shaking was maintained between successive readings.

**
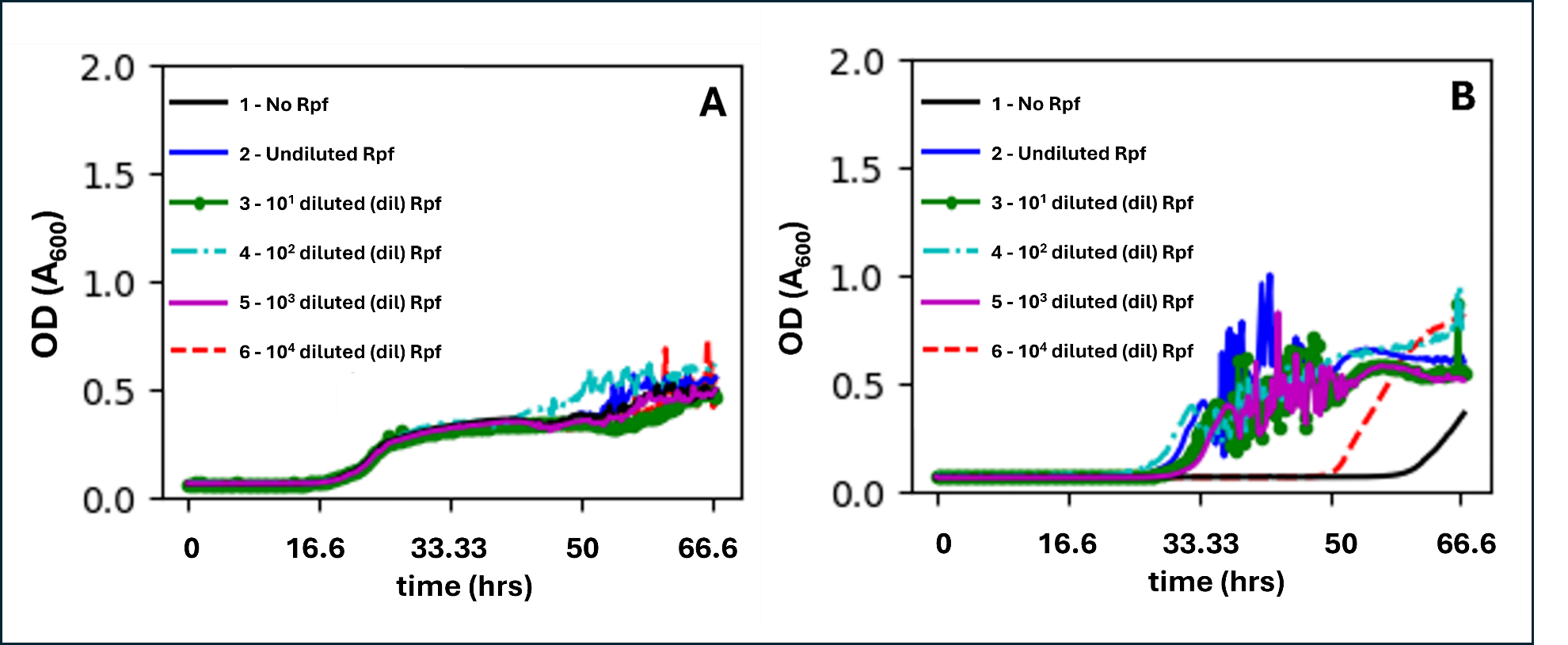
**

**Figure S8.** Nos 1-6 refers to column of cells in a 4x6 microtiter plate. A: Growth curve of *T. phoenicis* (obtained from logarithmic phase of growth in AMM) resuspended in AMM media. B: Growth curve of cells of *T. phoenicis* having been air-dried for 168 hrs (7 days), resuspended in water and used as an inoculum in AMM. In both rows, column 1 had no Rpf (lysate of *E. coli* overexpressing the Rpf gene from *M. luteus*). Column 2 had 10 µl of undiluted Rpf (lysate) ((~1µM (27.5 µg/ml)), and columns 3-6 serially diluted Rpf (lysate). Growth was conducted and measured every 15 minutes in a 24-well (4×6) microtiter plate with 1mL of medium per well using a Tecan SpectraFluor Plus instrument. Continuous shaking was maintained between successive readings.
